# *Campylobacter jejuni* Cytolethal Distending Toxin C Exploits Lipid Rafts to Mitigate *Helicobacter pylori*-Induced Pathogenesis

**DOI:** 10.3389/fcell.2020.617419

**Published:** 2021-02-23

**Authors:** Jia-Yin Yeh, Hwai-Jeng Lin, Chia-Jung Kuo, Chun-Lung Feng, Chia-Huei Chou, Chia-Der Lin, Hui-Yu Wu, Chen-Yi Li, Cheng-Hsun Chiu, Chih-Ho Lai

**Affiliations:** ^1^Department of Microbiology and Immunology, Graduate Institute of Biomedical Sciences, Chang Gung University, Taoyuan, Taiwan; ^2^Division of Gastroenterology and Hepatology, Department of Internal Medicine, Shuang-Ho Hospital, New Taipei, Taiwan; ^3^Division of Gastroenterology and Hepatology, Department of Internal Medicine, School of Medicine, College of Medicine, Taipei Medical University, Taipei, Taiwan; ^4^Chang Gung Microbiota Therapy Center, Department of Gastroenterology and Hepatology, Chang Gung Memorial Hospital, Linkou, Taiwan; ^5^College of Medicine, Chang Gung University, Taoyuan, Taiwan; ^6^Division of Gastroenterology and Hepatology, Department of Internal Medicine, China Medical University Hsinchu Hospital, Hsinchu, Taiwan; ^7^Department of Microbiology, School of Medicine, China Medical University, Taichung, Taiwan; ^8^Department of Infectious Disease, Department of Otolaryngology-Head and Neck Surgery, China Medical University Hospital, Taichung, Taiwan; ^9^Molecular Infectious Disease Research Center, Department of Pediatrics, Chang Gung Memorial Hospital, Linkou, Taiwan; ^10^Department of Nursing, Asia University, Taichung, Taiwan

**Keywords:** *Helicobacter pylori*, cytolethal distending toxin, lipid rafts, cytotoxin-associated gene A, vacuolating cytotoxin A

## Abstract

*Helicobacter pylori* infection is associated with several gastrointestinal diseases, including gastritis, peptic ulcer, and gastrointestinal adenocarcinoma. Two major cytotoxins, vacuolating cytotoxin A (VacA) and cytotoxin-associated gene A (CagA), interact closely with lipid rafts, contributing to *H. pylori*-associated disease progression. The *Campylobacter jejuni* cytolethal distending toxin consists of three subunits: CdtA, CdtB, and CdtC. Among them, CdtA and CdtC bind to membrane lipid rafts, which is crucial for CdtB entry into cells. In this study, we employed recombinant CdtC (rCdtC) to antagonize the functions of *H. pylori* cytotoxin in cells. Our results showed that rCdtC alleviates cell vacuolation induced by *H. pylori* VacA. Furthermore, rCdtC reduces *H. pylori* CagA translocation, which decreases nuclear factor kappa-B activation and interleukin-8 production, resulting in the mitigation of gastric epithelial cell inflammation. These results reveal that CdtC hijacks cholesterol to compete for *H. pylori* cytotoxin actions *via* lipid rafts, ameliorating *H. pylori*-induced pathogenesis.

## Introduction

*Helicobacter pylori* commonly colonizes the human stomach and infects approximately 50% of humans worldwide (Amieva and Peek, 2016). Persistent *H. pylori* infection may be associated with a high risk of gastrointestinal diseases, including chronic gastritis, peptic ulcers, and gastric cancer (Crowe, 2019). Combination therapies containing a proton pump inhibitor (PPI) and several antibiotics have been used to eradicate *H. pylori* for decades (Hentschel et al., 1993). Given the widespread use of antibiotics, *H. pylori* antimicrobial resistance rates have increased annually and is currently the main cause of treatment failure (Alba et al., 2017).

Several virulence factors are involved in *H. pylori*-induced pathogenesis and sustain infection in a host organism (Lu et al., 2005). Vacuolating cytotoxin A (VacA) and cytotoxin-associated gene A (CagA) are two major bacterial cytotoxins that contribute to *H. pylori*-related disease progression (Salama et al., 2013). VacA secreted by *H. pylori* binds to its receptors to form vesicles containing anion-selective channels, resulting in increased osmotic swelling, and vacuolation (Cover and Blanke, 2005). CagA, another crucial virulence factor, is injected into host cells *via* the *H. pylori* type IV secretion system (TFSS) (Hatakeyama, 2008; Herrera and Parsonnet, 2009). Translocated CagA is then phosphorylated, leading to NF-κB activation, IL-8 production (Brandt et al., 2005), and cell scattering (also referred to as the hummingbird phenotype) (Segal et al., 1999).

Lipid rafts are rigid microdomains located on the cell membrane, which comprise large proportions of phospholipids, sphingolipids, and cholesterol (Ikonen, 2001). Several studies have demonstrated that *H. pylori* VacA and CagA exploit lipid rafts as toxin receptors for their intoxication (Schraw et al., 2002; Lai et al., 2008; Murata-Kamiya et al., 2010). In addition, *H. pylori* cholesterol-α-glucosyltransferase catalyzes the conversion of cholesterol into cholesterol α-glucosides, which are crucial for bacterial evasion of the immune response (Wunder et al., 2006; Lai et al., 2018; Morey et al., 2018). *Campylobacter jejuni* cytolethal distending toxin (CDT), a genotoxin, contains three subunits: CdtA, CdtB, and CdtC (Lara-Tejero and Galan, 2000). Similar to many bacterial toxins, CdtA and CdtC serve as binding moieties and attach to cholesterol-rich microdomains that enhance CdtB delivery into cells (Lin et al., 2011).

Usurping or depleting cholesterol disrupts lipid rafts, which ameliorates *H. pylori*-induced inflammation and pathogenesis (Lin C. J. et al., 2016; Lin et al., 2017). The development of effective agents against VacA and CagA that interact with lipid rafts might be an ideal strategy for alleviating *H. pylori*-induced pathogenesis. Given the notion that CdtC contains a cholesterol recognition/interaction amino acid consensus (CRAC) domain that specifically binds to cholesterol in the membrane rafts (Lai et al., 2013), it is worth investigating whether CdtC has an inhibitory effect on *H. pylori* cytotoxin-induced pathogenesis. In this study, the biological functions of CdtC antagonizing VacA/CagA toxin actions in cells were extensively investigated. Our results reveal that *C. jejuni* CdtC hijacks cholesterol to compete with cytotoxin functions. Thus, *C. jejuni* CdtC can be potentially developed as a preventive agent against *H. pylori*-induced pathogenesis.

## Materials and Methods

### Cell and Bacterial Culture

Human gastric epithelial cells (AGS cells, ATCC CRL-1739) were cultured in F12 medium (Hyclone, Logan, UT, United States) containing 10% fetal bovine serum (Hyclone). The cells were maintained at 37°C containing 5% CO_2_. *H. pylori* strain 26695 (ATCC 700392) and CagA-EGFP *H. pylori* (Lin et al., 2013) were routinely cultured on blood agar plates (Brucella agar with 10% defibrinated sheep blood) and incubated at 37°C in a microaerophilic environment (5% O_2_, 10% CO_2_, and 85% N_2_). *Escherichia coli* strain BL21-DE3 (pET21d-*cdtC*) was cultured on LB agar plate containing 100 μg/ml ampicillin and incubated at 37°C as described previously (Lin et al., 2011).

### Preparation of Recombinant CdtC

Recombinant CdtC was constructed and characterized as described previously (Lin et al., 2019). Briefly, *E. coli* BL21-DE3 containing pET21d-*cdtC* was cultured and induced by isopropyl β-D-thiogalactopyranoside (1 mM) at 16°C for 4 h. The bacterial cells were lysed, and rCdtC protein was purified by metal affinity chromatography (Clontech, Palo-Alto, CA, United States). The purified rCdtC was characterized by SDS-PAGE and western blot assay (Supplementary Figure 1).

### Cell Survival Assay

AGS cells (2 × 10^4^) were cultured in 96-well plates for 16 h and incubated with rCdtC (200, 500, and 1,000 nM) at 37°C for 1, 7, and 11 h. The cells were then treated with 100 μl of 5 mg/ml 3-(4,5-dimethylthiazol-2-yl)–2,5-diphenyltetrazolium bromide (MTT) solution (Sigma-Aldrich, St. Louis, MO, United States) at 37°C for 2 h. The ability of viable cells to reduce MTT to formazan was measured (Chen Y. W. et al., 2020). Cell viability was expressed as fold changes compared to the untreated group.

### Bacterial Viability Assay

*Helicobacter pylori* was cultured for 30 h to reach OD_600_ of 1.0. The bacteria were treated with rCdtC (200, 500, and 1,000 nM) at 37°C for 6 h. The bacteria were then plated by serial dilution on blood agar plates. Colony-forming units (CFUs) were enumerated to determine the viable bacteria (Lien et al., 2019).

### Vacuolation Activity Assay

*Helicobacter pylori* VacA-induced cell vacuolation was assessed using neutral red uptake assay as described previously (Cover et al., 1991). Briefly, AGS cells (1 × 10^5^) were plated in 24-well plates and incubated with 200 nM rCdtC for 1 h, followed by *H. pylori* infection at a multiplicity of infection (MOI) of 100 for 6 h. The cells were washed with phosphate-buffered saline (PBS) and incubated with 0.05% neutral red (Sigma-Aldrich) for 4 min with gentle shaking. Afterward, acidified alcohol (1% 12 N HCl in 75% ethanol) was added to elute the solution, which was determined at OD_540_ by using a spectrophotometer (Molecular Devices, San Jose, CA, United States).

### Immunofluorescence Staining and Confocal Microscopic Analysis

AGS cells (3 × 10^5^) were seeded in six-well plates and cultured for 16 h. rCdtC (200 nM) was treated for 1 h prior to infection with wild-type or CagA-EGFP *H. pylori* at a MOI of 20 for 2 h. The cells were fixed in 4% paraformaldehyde (Alfa Aesar, Haverhill, MA, United States) for 1 h, probed with anti-CdtC antibody and anti-VacA antibody (Santa Cruz, Dallas, TX, United States), and then stained with Alexa Fluor 488-conjugated anti-mouse IgG (Jackson ImmunoResearch, West Grove, PA, United States), Cy5-conjugated anti-mouse IgG (Invitrogen, Carlsbad, CA, United States), or anti-goat IgG-conjugated CruzFluor 555 (Santa Cruz). The plasma membrane was probed with wheat germ agglutinin (WGA)-conjugated Alexa Flour 594 (Thermo Fisher Scientific, Waltham, MA United States). The nuclei were stained with Hoechst 33342 for 20 min. The stained cells were visualized using a confocal laser scanning microscope (LSM780, Carl Zeiss, Germany) and analyzed by the software ZEN (Carl Zeiss). VacA and CagA presented in the cytoplasm were quantified by green fluorescence intensity. Green puncta representing VacA and CagA existing in the bacteria were excluded from the fluorescence intensity analysis. The quantification was the mean pixel intensity of the VacA and CagA signal shown in the cytoplasm of each cell (50 cells were analyzed per sample).

### Western Blot Assay

The rCdtC-treated and *H. pylori*-infected cells were prepared and analyzed by 12% SDS-PAGE, followed by transferring them onto polyvinylidene difluoride membrane (Millipore, Burlington, MA, United States). The membrane was incubated with primary antibody and then incubated with horseradish peroxidase-conjugated secondary antibody (Millipore, Temecula, CA, United States). The protein expression level was detected using ECL western blotting detection reagents (GE Healthcare, Chicago, IL, United States) and analyzed by Azure 400 (Azure Biosystems, Dublin, CA, United States).

### Quantitation of Cells With Hummingbird Phenotype

AGS cells (3 × 10^5^) were seeded in six-well plates and cultured for 16 h. The cells were treated with rCdtC (200 and 500 nM) or 0.1 μM of bafilomycin A1 (BafA1) (InvivoGen, San Diego, CA, United States) for 1 h prior to *H. pylori* infection at a synchronized MOI of 100 for 6 h. The elongated cells (hummingbird phenotype) were defined as cells that showed thin needle-like protrusions of more than 20 μm in length and a typical elongated shape, as reported previously (Wang et al., 2012). The percentage of elongated cells was determined as the number of cells having the hummingbird phenotype.

### Luciferase Reporter Assay

AGS cells were transfected with NF-κB-luciferase reporter by jetPEI (Polyplus-transfection, France) as described previously (Lin H. J. et al., 2016). The cells were treated with 200 nM rCdtC for 1 h, followed by *H. pylori* infection at a MOI of 100 for 6 h. The cells were lysed, and luciferase assays were performed using the Dual-Luciferase Reporter Assay System (Promega, Madison, MA, United States) with a microplate luminometer (Biotek, Winooski, VT, United States). Luciferase activity was normalized for transfection efficiency by the co-transfected β-galactosidase expression vector (Promega).

### IL-8 Measurement

AGS cells (1 × 10^5^) were seeded in 24-well plates and cultured for 16 h. The cells were treated with 200 nM rCdtC for 1 h and then infected with *H. pylori* at a MOI of 100 for 6 h. The supernatant was prepared, and the IL-8 concentration was measured by using sandwich enzyme-linked immunosorbent (ELISA) assay according to the manufacturer’s instructions (Thermo Fisher Scientific).

### Gentamicin Protection Assay

AGS cells were treated with 200 nM rCdtC for 1 h prior to *H. pylori* infection at a MOI of 100 for 6 h. The treated cells were washed with PBS three times, followed by incubation with 100 μg/ml gentamicin (Sigma-Aldrich) for 90 min. The cells were lysed with sterile H_2_O for 10 min, and cell lysate was plated by serial dilution on blood agar plates. Viable CFUs were enumerated to determine the activity of *H. pylori* invasion of cells, as described previously (Chen et al., 2019).

### Statistical Analysis

The data were presented as mean ± standard deviation of triplicate independent experiments. Student’s *t*-test was performed to evaluate the statistical significance of the experimental results between two groups by using SPSS program (version 12.0 for Windows, SPSS Inc., Chicago, IL, United States). A *p*-value less than 0.05 was considered as statistically significant.

## Results

### Purification and Characterization of *C. jejuni* rCdtC

Recombinant CdtC was first purified and validated by SDS-PAGE and western blot analysis (Supplementary Figure 1). We then examined the influence of rCdtC on cell viability by treating AGS cells with various concentrations of rCdtC (200, 500, and 1,000 nM) for 1, 7, and 11 h. As shown in Supplementary Figure 2A, cell viability was barely affected by the treatment doses. In addition, incubation of *H. pylori* with rCdtC for 6 h only resulted in a marginal influence on bacterial survival at the highest dose (1,000 nM) (Supplementary Figure 2B). These results indicate that rCdtC neither influences cell viability nor affects *H. pylori* survival.

### rCdtC Reduces *H. pylori*-Induced Vacuolation in Gastric Epithelial Cells

**FIGURE 1:**
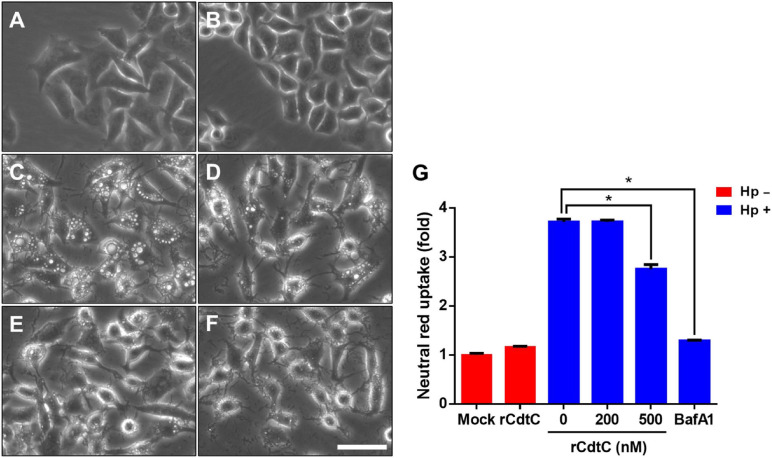
rCdtC inhibits *Helicobacter pylori*-induced vacuolation in gastric epithelial cells. AGS cells were **(A)** untreated with rCdtC, **(B)** treated with 200 nM rCdtC alone, **(C)** infected with *H. pylori* for 6 h, **(D)** pretreated with 200 nM rCdtC prior to *H. pylori* infection for 6 h, **(E)** pretreated with 500 nM rCdtC followed by *H. pylori* infection for 6 h, and **(F)** pretreated with 0.1 μM BafA1 then infected with *H. pylori* for 6 h. Cell vacuolation was observed by using a phase-contrast microscope. Scale bar, 100 μm. **(G)** Neutral red uptake assay was employed to analyze the vacuolating activity. Each group was performed in three independent experiments (*P < 0.05).

**FIGURE 2:**
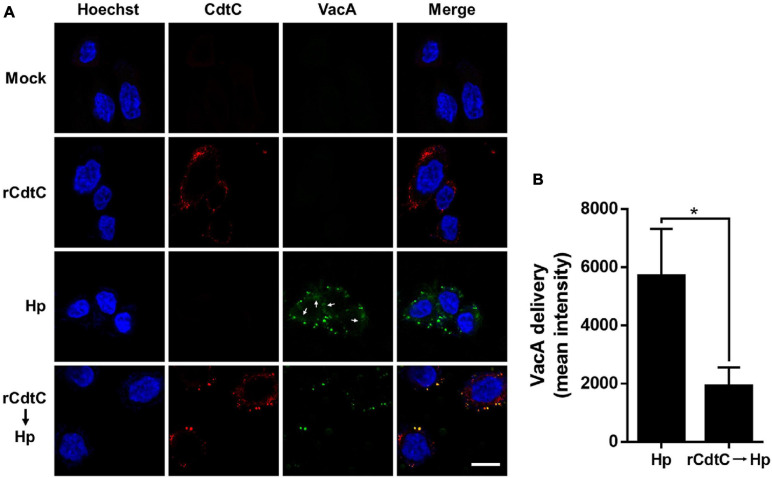
rCdtC declines *Helicobacter pylori* VacA delivery in cells. AGS cells **(A)** pre-incubated with rCdtC (200 nM) were infected with or without *H. pylori* at a multiplicity of infection of 100 for 6 h. The cells were stained with anti-CdtC (red) and anti-VacA (green), and the nuclei were probed with Hoechst 33342 (blue). The image was observed under a confocal microscope with a 63× oil immersion objective and processed by software ZEN (Carl Zeiss, Germany). Arrows indicate VacA delivered into the cytoplasm. Scale bar, 10 μm. **(B)** The delivery of VacA into the cytoplasm was quantified. Green puncta representing VacA localized on the bacterial surface were excluded from the fluorescence intensity analysis. The quantification represented in the figures reflected the mean pixel intensity of the VacA signal in the cytoplasm of each cell (50 cells were analyzed per sample). *P < 0.05.

*Helicobacter pylori* VacA assembles on the cell membrane and is delivered intracellularly by exploiting cholesterol-rich lipid rafts (Schraw et al., 2002; Gupta et al., 2008). Binding of *C. jejuni* CdtC to membrane cholesterol is a crucial step for CDT entry into cells (Lai et al., 2013, 2015). Considering that membrane cholesterol is essential for CdtC binding and VacA delivery, we investigated whether rCdtC hijacks cholesterol to compete with VacA actions in cells. AGS cells were untreated (Figure 1A) or pretreated with rCdtC (Figures 1B,D,E) or bafilomycin A1 (BafA1) (Figure 1F) for 1 h, followed by *H. pylori* infection for 6 h. As shown in Figure 1C, vacuole formation in *H. pylori*-infected cells was noticeable when compared to the mock control. Treatment of cells with rCdtC (500 nM) or BafA1 (0.1 μM) markedly inhibited *H. pylori*-induced vacuolation in cells (Figure 1G). The intracellular delivery efficiency of VacA was then investigated using confocal microscopy. As shown in Figure 2, *H. pylori*-infected cells exhibited VacA in the cytoplasm. Pretreatment of cells with rCdtC significantly reduced intracellular VacA delivery. These results demonstrate that rCdtC possesses inhibitory activity against *H. pylori* VacA actions in gastric epithelial cells.

### rCdtC Suppresses *H. pylori* CagA-Induced Pathogenesis in Gastric Epithelial Cells

**FIGURE 3:**
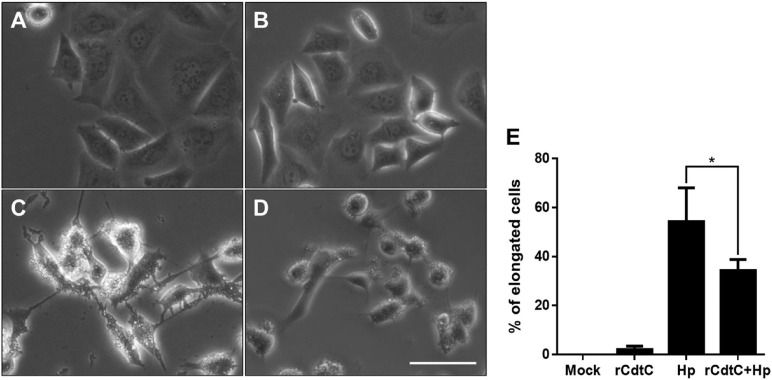
rCdtC attenuates *Helicobacter pylori*-induced cell scattering. AGS cells were **(A)** untreated with rCdtC, **(B)** treated with 200 nM rCdtC alone, **(C)** infected with *H. pylori* for 6 h, and **(D)** pretreated with rCdtC then infected with *H. pylori* for 6 h. Scale bar, 100 μm. **(E)** The elongated cells were counted and expressed as percentage compared to the untreated cells. The results were performed in three independent experiments and represented as mean ± SD (*P < 0.05).

**FIGURE 4:**
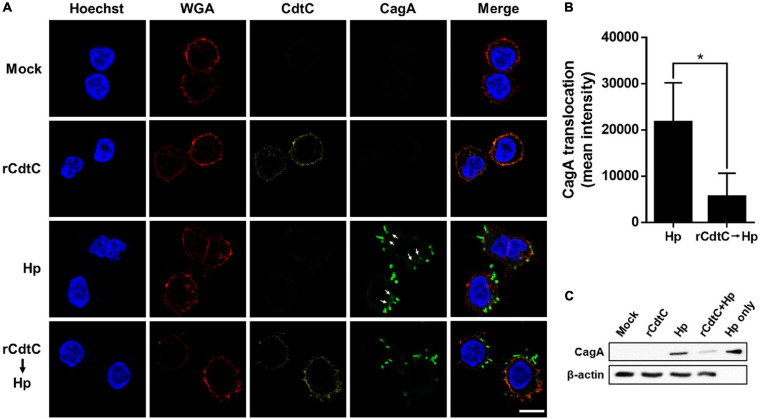
rCdtC decreases CagA translocation in *Helicobacter pylori*-infected cells. **(A)** AGS cells pre-incubated with rCdtC (200 nM) were infected with or without CagA-EGFP *H. pylori* (green) at a multiplicity of infection of 20 for 2 h. The cells were probed with anti-CdtC (yellow), the plasma membrane was stained with WGA-conjugated Alexa Flour 594 (red), and the nuclei were stained with Hoechst 33342 (blue). Arrows indicate CagA translocated into the cytoplasm. The image was analyzed by a confocal microscope with 63× oil immersion objective and processed by software ZEN. Scale bar, 10 μm. **(B)** CagA-EGFP translocation was quantified by green fluorescence intensity. Green puncta representing CagA that existed in the bacteria were excluded from the fluorescence intensity analysis. The quantification indicated the mean pixel intensity of the CagA signal in the cytoplasm of each cell (50 cells were analyzed per sample). *P < 0.05. **(C)** The level of CagA translocation was determined by using western blot assay. β-Actin expression was used to represent an internal control for equal loading.

*Helicobacter pylori* CagA translocation is important for cytoskeleton rearrangement, leading to cell elongation and scattering (hummingbird phenotype) (Segal et al., 1999). We then evaluated whether rCdtC influences cell scattering in *H. pylori*-infected cells. As shown in Figure 3C, the hummingbird phenotype appeared in *H. pylori*-infected cells, but not shown in cells untreated with rCdtC (Figure 3A) and treated with rCdtC along (Figure 3B). However, the *H. pylori*-induced elongated phenotype was significantly inhibited in the cells pretreated with rCdtC (Figures 3D,E). We further analyzed the inhibition of CagA translocation by rCdtC using confocal microscopy. As shown in Figure 4A, WGA was used to label the plasma membrane. Infection of cells with CagA-EGFP *H. pylori* led to prominent CagA translocation to the cytoplasm (Figures 4A,B). Noticeably, less CagA translocation was observed in cells treated with rCdtC, followed by *H. pylori* infection (Figure 4C). We then investigated the distribution of rCdtC and CagA in cells by confocal microscopy. As shown in Supplementary Figure 3, the area across the cell membrane is represented by the peak of WGA fluorescence intensity. In the absence of rCdtC treatment, CagA-EGFP showed cytoplasmic distribution. In contrast, cells pretreated with rCdtC exhibited low CagA-EGFP signal in the cytoplasm. It can be noted from Supplementary Figure 3 that the rCdtC fluorescence intensity overlaps with the WGA signal, indicating the colocalization of rCdtC to the cell membrane. These results demonstrate that rCdtC binds to the cell membrane and inhibits CagA translocation, leading to the alleviation of CagA-induced pathogenesis.

### rCdtC Attenuates *H. pylori*-Induced Inflammation in Gastric Epithelial Cells

**FIGURE 5:**
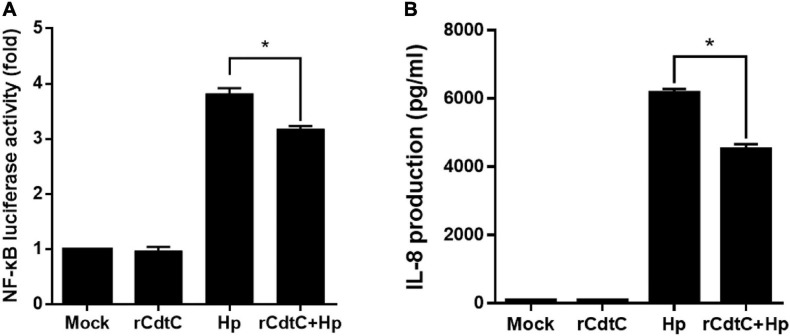
rCdtC suppresses NF-κB activation and IL-8 production in *Helicobacter pylori*-infected cells. AGS cells were untreated with rCdtC (mock), treated with 200 nM rCdtC, infected with *H. pylori* at a multiplicity of infection of 100 for 6 h, or pretreated with rCdtC then infected with *H. pylori*. **(A)** The level of NF-κB activation was determined by luciferase assay. **(B)** IL-8 production was assessed using ELISA. Each group was performed in triplicate experiments (*P < 0.05).

Since CagA translocation leads to NF-κB activation and IL-8 production enhancement in AGS cells (Brandt et al., 2005), we sought to investigate whether rCdtC affects IL-8 production in *H. pylori*-infected cells. As shown in Figure 5A, *H. pylori* infection induced higher NF-κB luciferase activity than mock or rCdtC treatment alone. However, in cells pretreated with rCdtC, *H. pylori*-induced NF-κB activation was markedly diminished compared to the rCdtC-untreated group. Similar to the results of NF-κB activation, we observed an inhibitory effect on IL-8 production (Figure 5B). These results demonstrate that rCdtC inhibits CagA-associated NF-κB activation and IL-8 secretion, and it subsequently ameliorates *H. pylori*-induced inflammation.

### rCdtC Reduces *H. pylori* Invasion of Cells

**FIGURE 6:**
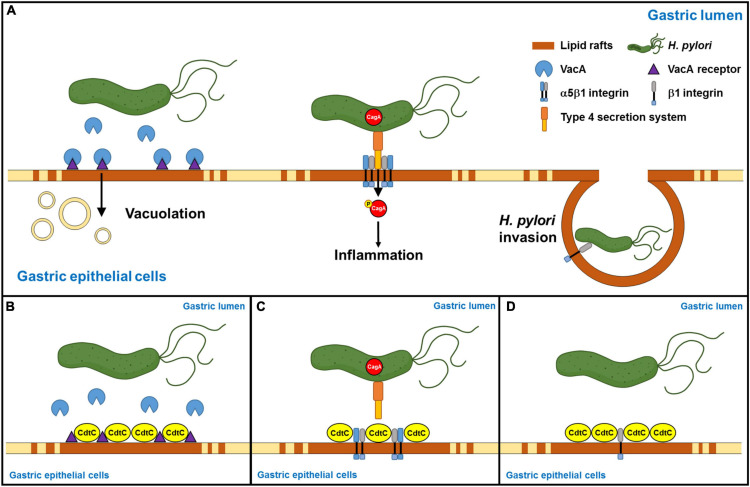
Schematic representation of this study to summarize *Campylobacter jejuni* CdtC action and its potential application in the alleviation of *Helicobacter pylori*-induced pathogenesis. **(A)** *H. pylori*-secreted VacA binds its receptors in the lipid rafts, followed by inducing vacuolation formation in the cells. Furthermore, *H. pylori* directly injects CagA into the host cells through the interaction of TFSS and cellular α5β1 integrin in the lipid rafts. Then, CagA is phosphorylated, which caused an inflammatory response. Subsequently, *H. pylori* invasion depends on *H. pylori* interacting with β1 integrin. **(B)** *C. jejuni* CdtC inhibits VacA-induced cell vacuolation, **(C)** suppresses CagA translocation, and **(D)** declines *H. pylori* invasion in the gastric epithelial cells through the binding of lipid rafts.

*Helicobacter pylori* internalized by gastric epithelial cells requires membrane rafts, whereas adhesion does not (Lai et al., 2008). Our results showed that rCdtC did not inhibit *H. pylori* adhesion in AGS cells (Supplementary Figure 4A). We then examined whether rCdtC affects *H. pylori* invasion in cells using the gentamicin protection assay. As expected, rCdtC effectively reduced *H. pylori* invasion in cells (Supplementary Figure 4B). These results indicate that rCdtC decreases *H. pylori* invasion of cells but has no impact on *H. pylori* adhesion to cells. Collectively, our results reveal that rCdtC binding to membrane rafts competes with the interactions between cholesterol-rich microdomains and bacterial actions, including VacA delivery, CagA translocation, and bacterial internalization in gastric epithelial cells, alleviating *H. pylori*-induced pathogenesis (Figure 6).

## Discussion

The gold-standard method for treating *H. pylori*-infected patients consists of PPI and several antibiotics, and it has been used as a first-line regimen for over two decades (Malfertheiner et al., 2017). Although antibiotic therapy showed marked antimicrobial efficacy in the beginning, the regimen is gradually becoming ineffective due to the rising antibiotic resistance of *H. pylori* (Alba et al., 2017). Particularly, antibiotic treatment may alter gut microbiota composition, which plays important roles in multiple human physiological processes, where dysbiosis may lead to disease development (Vangay et al., 2015). Considering the many adverse effects of antibiotics, a new therapeutic modality that differs from antibiotics is urgently needed to combat *H. pylori*-associated diseases.

Pathogens and their virulence factors exploiting lipid rafts to gain entry into host cells have been reported elsewhere (Wang and Hajishengallis, 2008; Frisan, 2016; Chen Y. et al., 2020). Previous studies have used cholesterol-depleting agents to reduce pathogen infections by inhibiting their entry into target cells. For example, depletion of membrane cholesterol by methyl-β-cyclodextrin impaired pathogen attachment to the cell surface (Guo et al., 2017; Owczarek et al., 2018; Chen Y. et al., 2020). Statins, inhibitors of HMG-CoA reductase, are cholesterol-lowering agents that have been employed to reduce microbial infectivity (Boyd et al., 2012; Motzkus-Feagans et al., 2012; Skerry et al., 2014). Consistent with the previous reports, our recent studies also revealed that statin use decreased *H. pylori* infection and reduced the incidence of *H. pylori*-associated diseases (Lin C. J. et al., 2016; Lin et al., 2017; Liao et al., 2017). These lines of evidence indicated that pharmaceutical usurping or depletion of cholesterol agents that disrupt lipid rafts may pave the way for treating microbial infections.

We previously reported that CdtA and CdtC coalesce in lipid rafts, which is essential for CdtB delivery in cells (Lin et al., 2011). We then demonstrated that the CRAC motif in CdtC is required for cholesterol binding (Lai et al., 2013). However, the actual actions of CdtC binding to cholesterol-rich microdomains, which prevent lipid raft-mediated toxin functions, have never been studied. The present study showed that rCdtC did not affect cell viability, indicating that the rCdtC that we used in mammalian cells was reliable. Therefore, it is reasonable to investigate whether rCdtC can gain the potency to compete with lipid raft-mediated toxin actions, particularly in the initial step of bacterial toxin binding to the cell membrane.

Vacuolating cytotoxin A, one of the most crucial virulence factors produced by *H. pylori*, has been extensively explored. Previous epidemiological studies have indicated that *H. pylori-*containing toxigenic *vacA* alleles are closely associated with a high risk of severe gastrointestinal diseases, such as peptic ulcer disease and gastric adenocarcinoma (Kidd et al., 2001). In addition, VacA is recognized as a multifunctional toxin that targets various cell types, including gastric epithelial cells, parietal cells, and immune cells (Cover and Blanke, 2005). VacA possesses pore-forming activity, which is associated with *H. pylori*-induced disease severity (Palframan et al., 2012). Deletion of the membrane-associated region of VacA inhibits vacuole formation in cells, which subsequently prevents toxin-induced pathogenesis (Foo et al., 2010). In line with previous findings, our results demonstrated that rCdtC has an inhibitory effect on *H. pylori*–VacA functions, including VacA delivery and intracellular vacuolation. The molecular mechanism for rCdtC diminishing *H. pylori*-induced vacuolation in gastric epithelial cells is through the competition for VacA binding to membrane rafts at the initial step of toxin entry.

Cytotoxin-associated gene A translocation is mediated by TFSS, which is located in cholesterol-rich microdomains (Lai et al., 2008, 2011; Murata-Kamiya et al., 2010; Lin C. J. et al., 2016). The translocated CagA is then phosphorylated, inducing a scattering phenotype and elevating IL-8 secretion from gastric epithelial cells (Lai et al., 2008). Membrane lipid phosphatidylserine plays a key role in the delivery of CagA in cells (Murata-Kamiya et al., 2010; Tohidpour et al., 2017). Interestingly, the reduction of cellular cholesterol by statins decreases CagA translocation/phosphorylation, which reduces the risk of *H. pylori*-associated gastric cancer (Lin C. J. et al., 2016). Our current study shows that rCdtC binding to membrane rafts avoids CagA action in cells by prohibiting CagA translocation into cells, attenuating NF-κB activation, and decreasing IL-8 production. These lines of evidence indicate that raft therapeutics may be a feasible approach to prevent *H. pylori* CagA-related pathogenesis.

Lipid rafts can be used as a platform to efficiently deliver *H. pylori* virulence factors, including VacA and CagA. In addition, lipid rafts also serve as a gateway for *H. pylori* internalization and multiplication in cells (Sit et al., 2020). Therefore, it is worth developing therapeutic agents against lipid rafts that impede *H. pylori* infection in its initial steps. Although rCdtC exerts inhibitory effects on VacA/CagA functions as studied using cell-based models, the detailed mechanism underlying pharmacological signaling remains to be clarified. It is therefore crucial to examine rCdtC activity using animal or *in vivo* studies, which may provide substantial evidence for combating *H. pylori* infection.

## Conclusion

In summary, this study provides a cell-based platform to determine whether rCdtC antagonizes *H. pylori*-induced pathogenesis by binding to lipid rafts. Our results demonstrate that rCdtC inhibits cholesterol-mediated VacA delivery and vacuolation in the cytoplasm. Furthermore, binding of rCdtC to membrane rafts significantly restricted CagA translocation, followed by attenuated CagA-mediated pathogenesis. The interaction of CdtC with cholesterol-rich microdomains is likely to contribute to interference with *H. pylori* cytotoxin actions, thereby decreasing their toxicity in cells. These results suggest that the inhibition of membrane raft-mediated toxin functions might be a rational target for the development of novel agents to alleviate *H. pylori*-induced pathogenesis.

## Data Availability Statement

The original contributions presented in the study are included in the article/Supplementary Material, further inquiries can be directed to the corresponding authors.

## Author Contributions

C-HsC and C-HL contributed to the conception or design of this work. J-YY, H-JL, C-JK, C-LF, and C-HuC conducted the experimental study. C-DL, H-YW, and C-YL contributed to data analysis and interpretation. J-YY, H-JL, C-JK, C-HsC, and C-HL contributed to the writing of the manuscript. All authors gave final approval.

## Conflict of Interest

The authors declare that the research was conducted in the absence of any commercial or financial relationships that could be construed as a potential conflict of interest.

## Supplementary Material

Supplementary Figure 1 Purification and characterization of *Campylobacter jejuni* rCdtC. rCdtC was purified and analyzed by **(A)** SDS-PAGE and **(B)** western blot assay. Marker of molecular weight in kDa is shown on the left.

Supplementary Figure 2 rCdtC neither affects cell survival nor influences bacterial viability. **(A)** AGS cells were treated with rCdtC (200, 500, and 1,000 nM) for 1, 7, and 11 h, and cell viability was assessed by 3-(4,5-dimethylthiazol-2-yl)-2,5-diphenyltetra-zolium bromide assay. **(B)** *Helicobacter pylori* was exposed to rCdtC (200, 500, and 1,000 nM) for 6 h. Bacteria grown on the blood agar plates were counted and defined as *H. pylori* viability.

Supplementary Figure 3 rCdtC suppresses CagA translocation of *Helicobacter pylori*-infected cells. **(A)** AGS cells pre-incubated with rCdtC (200 nM) were infected with CagA-EGFP *H. pylori* (green) at a multiplicity of infection of 20 for 2 h. The cells were probed with anti-CdtC (yellow), the plasma membrane was stained with WGA-conjugated Alexa Flour 594 (red), and the nuclei were stained with Hoechst 33342 (blue). The image was observed under a confocal microscope with × 63 oil immersion objective and processed by software ZEN. Arrows indicate the CagA translocated into the cytoplasm. Scale bar, 5 μm. **(B)** The distribution of fluorescence intensity for CdtC (yellow line), CagA (green line), and plasma membrane (red) signals across the white lines was calculated and presented as line intensity histograms in the right panels. The area within the cell membrane is represented by the peak of wheat germ agglutinin fluorescence intensity, which is located between the two dotted lines.

Supplementary Figure 4 rCdtC decreases *Helicobacter pylori* invasion in gastric epithelial cells. AGS cells were untreated with rCdtC (mock), treated with 200 nM rCdtC, infected with *H. pylori* for 6 h, and pretreated with rCdtC then infected with *H. pylori* for 6 h. *H. pylori* **(A)** adhesion and **(B)** invasion to cells were analyzed. **P* < 0.05.
