# Supplementary figures and images for: Campylobacter jejuni Cytolethal Distending Toxin C Exploits Lipid Rafts to Mitigate Helicobacter pylori-Induced Pathogenesis
Source: Front Cell Dev Biol. 2021 Feb 23;8:617419. doi: 10.3389/fcell.2020.617419 (PMC7940356; doi:10.3389/fcell.2020.617419)

**Figure S1**

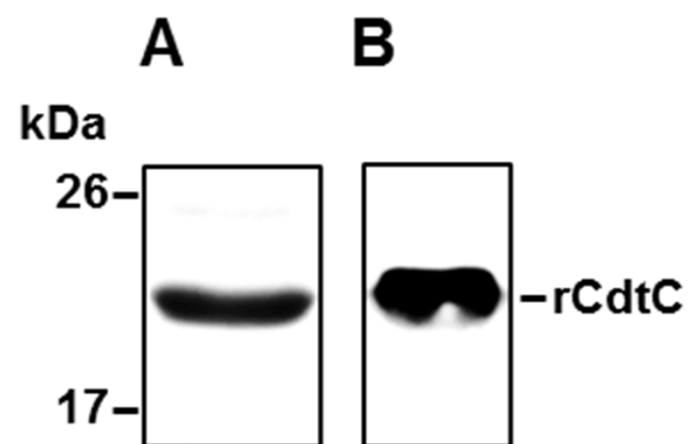

**Figure S2**

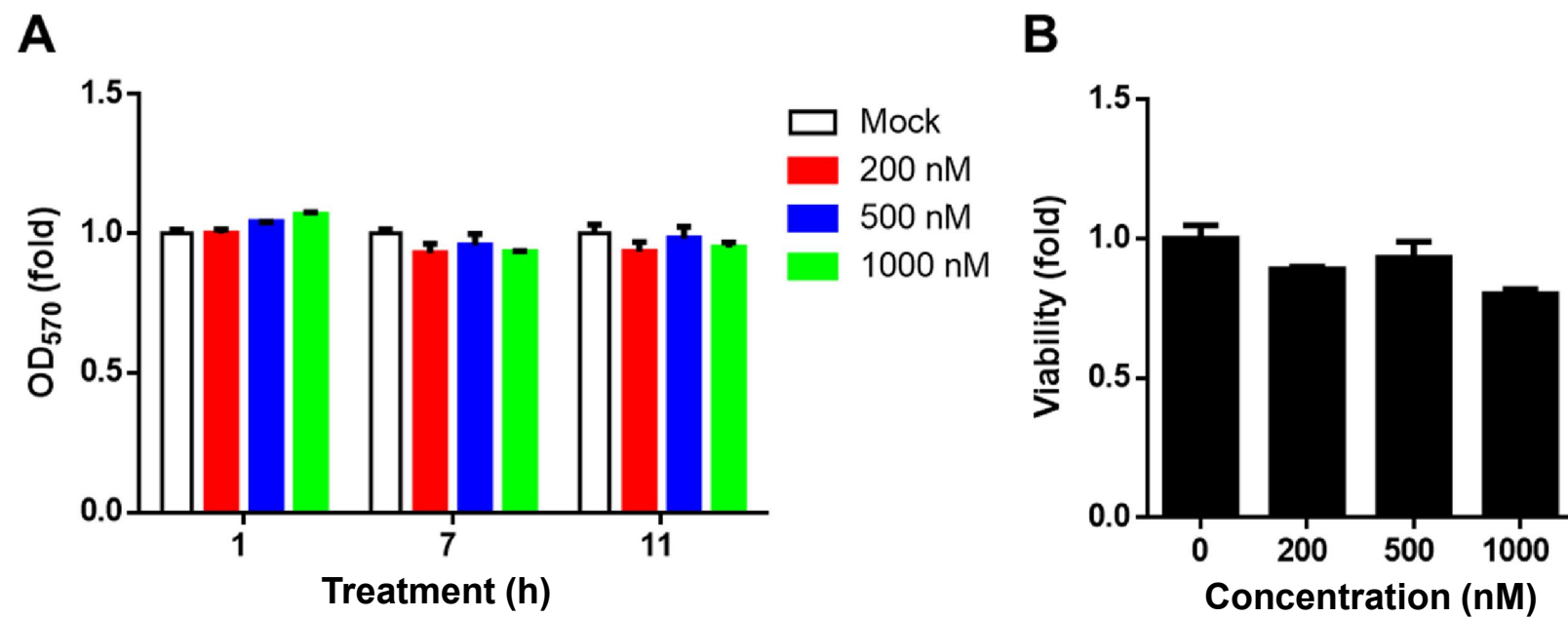

**Figure S3**

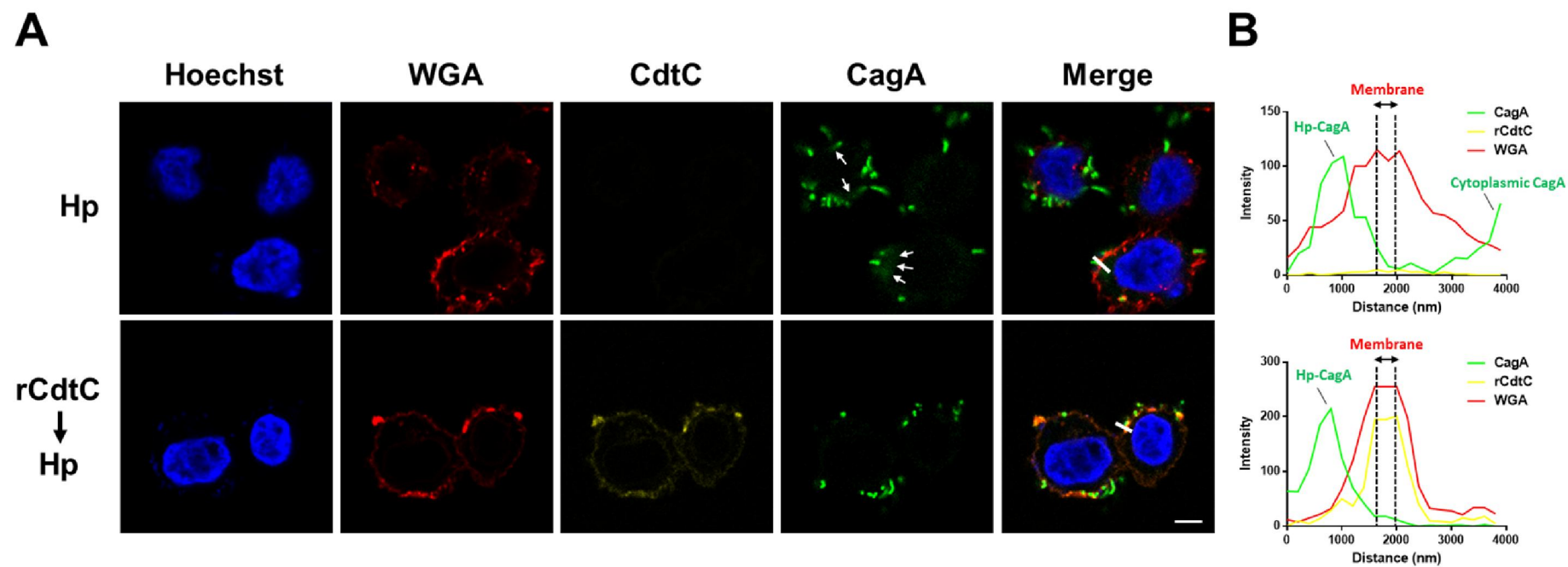

**Figure S4**

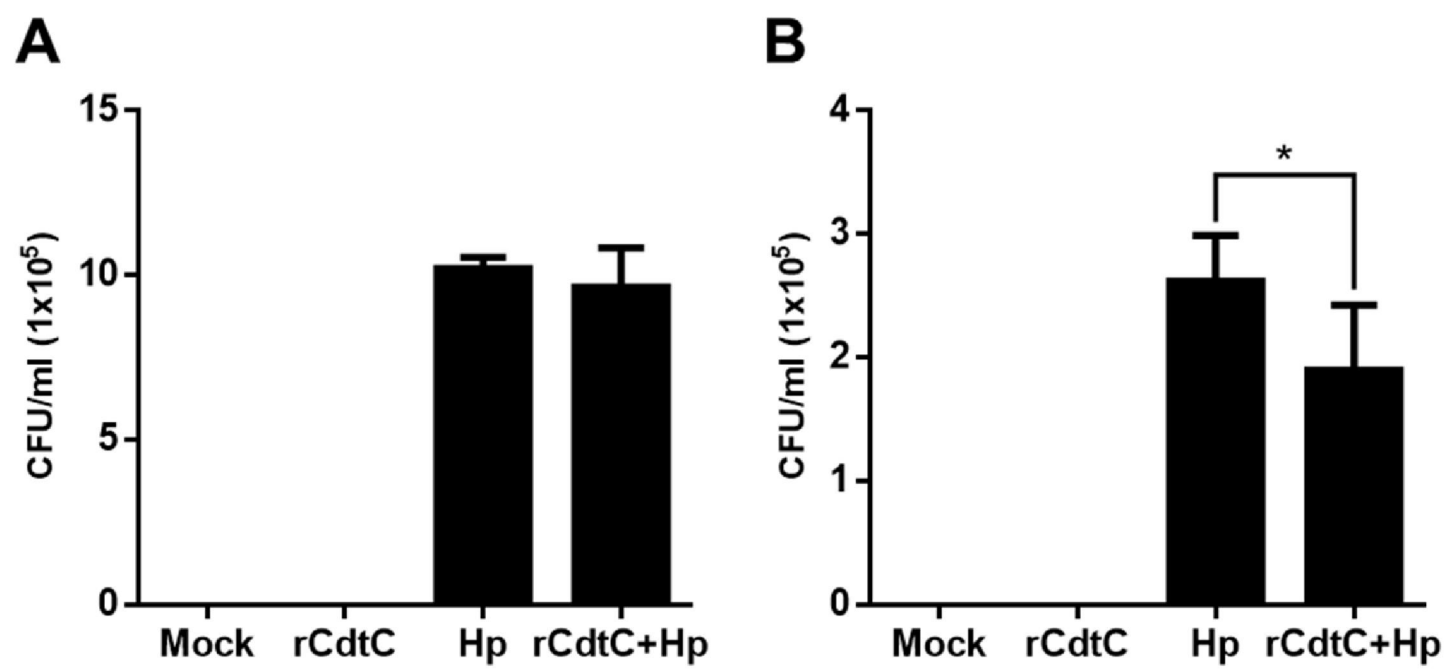

Supplement: Supplementary Figure 1 — Purification and characterization of Campylobacter jejuni rCdtC. rCdtC was purified and analyzed by (A) SDS-PAGE and (B) western blot assay. Marker of molecular weight in kDa is shown on the left. [file Data_Sheet_1.PDF]
